# A 3-year evaluation of preclinicaltrials.eu reveals room for improvement in preregistration of animal studies

**DOI:** 10.1371/journal.pbio.3001397

**Published:** 2021-09-09

**Authors:** Mira van der Naald, Steven A. J. Chamuleau, Julia M. L. Menon, Wim de Leeuw, Judith J. de Haan, Dirk J. Duncker, Kimberley E. Wever

**Affiliations:** 1 Department of Cardiology, University Medical Center Utrecht, Utrecht, the Netherlands; 2 Department of Cardiology, Amsterdam University Medical Center, Amsterdam, the Netherlands; 3 Netherlands Heart Institute, Utrecht, the Netherlands; 4 Animal Welfare Body Utrecht, Utrecht, the Netherlands; 5 Open Science Programme Utrecht, Utrecht University, Utrecht, the Netherlands; 6 Department of Cardiology, Thorax Center, Erasmus University Medical Center, Rotterdam, the Netherlands; 7 Systematic Review Centre for Laboratory animal Experimentation (SYRCLE), Department for Health Evidence, Nijmegen Institute for Health Sciences, Radboud University Medical Center, Nijmegen, the Netherlands

## Abstract

This Community Page article presents a 3-year evaluation of the first platform dedicated to preregistration of animal studies, www.preclinicaltrials.eu, and comparable platforms, encouraging the scientific community to embrace preregistration in a move towards more effective animal research.

## Teaser

In 2018, the first registry dedicated to preregistration of animal study protocols was launched. Despite international support, the overall number of (pre)registered protocols is still low, illustrating the need for pushing the preregistration agenda among researchers and policymakers.

## Why preregistration benefits research

Reporting of animal studies is poor, including incomplete reporting of study details, measures to reduce bias, and statistical analyses [1]. Poor reporting obscures the true state of affairs in animal studies, rendering external validity, internal validity, and statistical robustness and power largely unclear. The limitations found within studies are further exacerbated by reporting biases such as publication bias and selective outcome reporting. The publication rate of animal studies is limited to 60% to 67%, and, especially, studies yielding “negative” results remain unpublished [2–4]. Simultaneously, the underreporting of the number of animals used in publications suggests that data are reported selectively, an indication of outcome reporting bias [2]. Outcome switching and HARKing (hypothesising after results are known) are additional forms of bias known to be present in clinical research, and animal research is unlikely to be immune to these biases.

Registration of a protocol before starting an experiment (preregistration) plays an essential role in improving robustness and transparency of animal studies and leads to more reliable results. Such preregistration of preclinical studies has 4 main benefits:

1. disclosing the a priori study intention, i.e., hypothesis, exploratory or confirmatory character, and key elements of its design, including primary and secondary outcomes and sample size calculations;
2. promoting the use of methods to reduce risks of bias (i.e., blinding and randomisation) and creating transparency about their use;
3. providing a complete overview of all performed studies (including those that remain unpublished) and the possibility to share or link to related data; and
4. creating transparency and accountability within the research community and towards society.

## The development of preclinicaltrials.eu

In 2018, we launched the first online registry dedicated to animal study protocols: www.preclinicaltrials.eu [5]. The Netherlands Heart Institute was attracted as an independent party responsible for hosting and reviewing submitted protocols and the University Medical Center Utrecht (UMCU) formed the legal entity. An advisory board was established to advise the steering committee regarding, e.g., the future direction and implementation strategy of the registry. Discussions with several researchers and members of animal welfare bodies helped to optimise the registration form and identify concerns about preregistration. Common concerns were (1) administrative burden and additional costs; (2) limited flexibility of creativity; (3) misuse by animal activists; and (4) the fear of data theft/threats to intellectual property. Firstly, we aimed to determine the minimal amount of detail required to have an impact on research rigour, thereby minimising the additional administrative burden for researchers. We simultaneously set out to further reduce the administrative burden by enabling an automatic transfer of the required information from local digital systems to the preclinicaltrials.eu format. Within the UMCU, researchers will be able to copy most of the required information from their local application form to preclinicaltrials.eu with the click of a button. Once published, protocols can be amended, but all previous versions remain accessible. Regarding privacy, personal details of the researchers are anonymised, except for the institution where the experiments are performed. It is possible to contact the submitting researcher through an encrypted email message to facilitate collaboration. Detailed information of protocols can only be accessed after creating an account and logging in. Without an account, only the title and study centre details of protocols are visible. Regarding the fear of sharing preliminary ideas, preclinicaltrials.eu provides the option to register a protocol under embargo. The full details of the protocol remain hidden until revealed by the investigator or after a release date, which is automatically set at 1 year after registration.

Even though these concessions delay our aim to create full transparency, we feel that the benefits of preregistration outweigh this downside. Also, we propose that these solutions are necessary at this stage, until preregistration becomes the gold standard and the research community comes to view preregistration as a safeguard against intellectual theft of scientific ideas and intellectual property (since preregistration in fact “claims” an idea), rather than a risk.

## Results after 3 years of preclinicaltrials.eu

**Fig 1:**
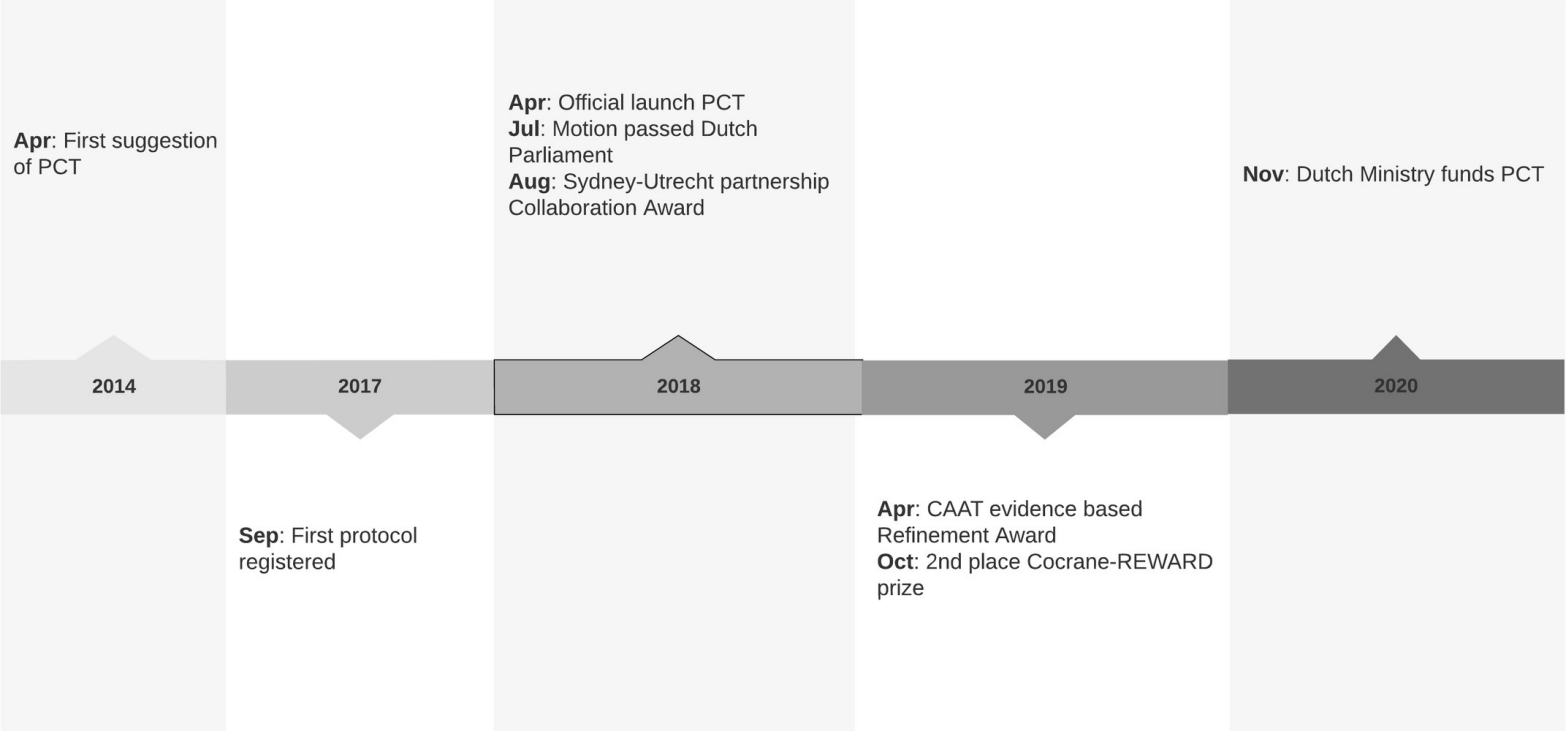
Timeline of the development of PCT. CAT, Center for Alternatives to Animal Testing; PCT, preclinicaltrials.eu.

In the Netherlands, the discussion on preregistration has intensified substantially over recent years (Fig 1). The Royal Netherlands Academy of Arts and Sciences stated in 2018 that funders and journals should make preregistration mandatory for hypothesis-testing research [6]. The Dutch House of Representatives unanimously accepted a motion stimulating preregistration for all animal research in the Netherlands [7]. In response, the Dutch government supported preclinicaltrials.eu, and the Dutch Ministry of Agriculture, Nature and Food Quality provided funding for its maintenance and further development [8,9]. The board of directors of the UMCU agreed to stimulate preregistration of animal studies within its facility, focusing primarily on preregistration of confirmatory studies. Several funding agencies (including the Collaborating Health Foundations) within the Netherlands support preregistration, and the Netherlands Organisation for Health Research and Development (ZonMw) made preregistration a requirement for funding of animal studies in several pilot programmes [10]. On an international level, preclinicaltrials.eu received prizes by among others the Johns Hopkins University Center for Alternatives to Animal Testing and the Cochrane-REWARD community.

**Fig 2:**
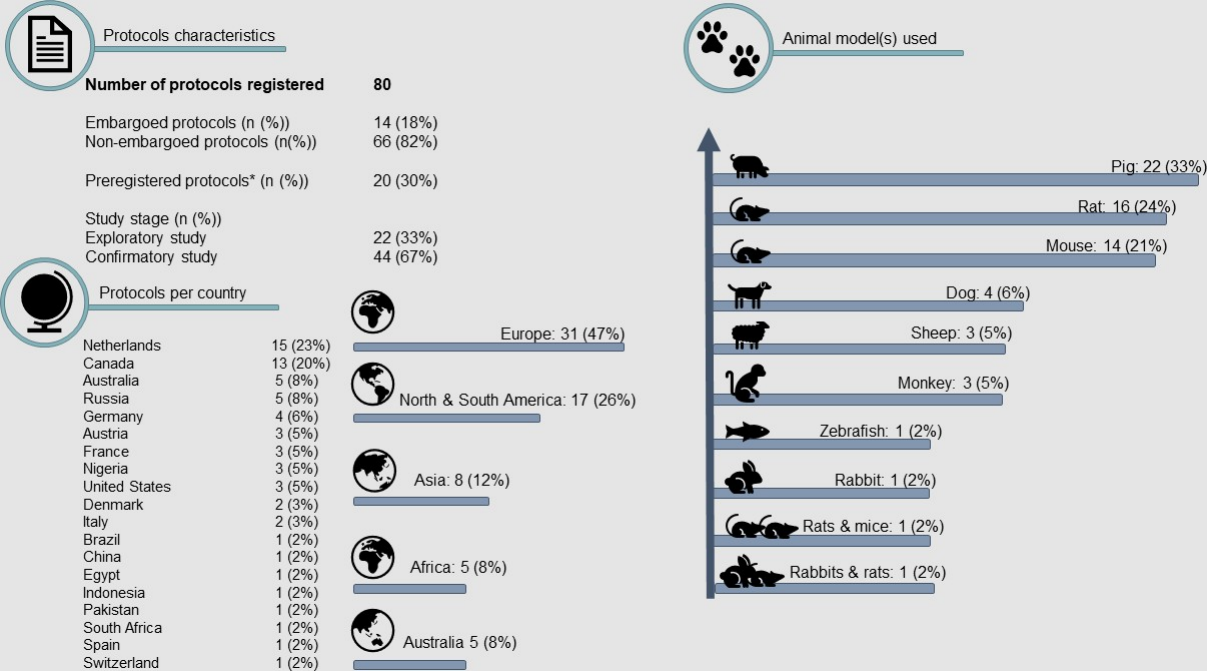
Protocols published on preclinicaltrials.eu on July 26, 2021. Note that only details of non-embargoed protocols are shown.* Preregistration is based on the reported study status at the first version of the submitted protocol.

Despite international recognition, the number of registered protocols is still low. As of July 26, 2021, 80 protocols have been registered, of which 14 are embargoed. The 66 non-embargoed protocols originate from 19 countries. They consist of both small animal (*n* = 34, 52%) and large animal (*n* = 32, 48%) studies. Most studies (*n* = 44, 66%) are confirmatory studies (Fig 2). Less than one-third of studies was registered before the start of the study (*n* = 20, 31%). Of note, the German Centre for the Protection of Laboratory Animals (Bf3R) launched a comparable platform for registration of animal studies (www.animalstudyregistry.org). This registry contains 87 studies from 12 different countries. Similarly, a low percentage of these studies was preregistered (*n* = 13, 15%). Most studies are embargoed (*n* = 73, 84%). Of the non-embargoed protocols, 3 studies (21%) have a confirmatory character, and 2 (14%) involve large animal models. Other preregistration platforms exist, but they are not free of charge or are not tailored to animal research. Considering that globally, over 58 million animals are used for scientific purposes yearly, the total of 167 protocols registered on the 2 dedicated animal study platforms over the last 3 years indicate that there is ample room for improvement [11].

## The future of preclinical preregistration

We believe it is time for the scientific community to take responsibility and move towards more effective animal research. We encourage funders to guard quality in research by making preregistration mandatory and journals to make preregistration a requirement for publication, akin to the standard for clinical trials. Approval committees and institutions involved in animal research should require accountability of previously provided animals as part of a new application, and funders could do the same for new funding applications. Institutes can incorporate preregistration in their reward system, and journals can reward researchers who preregister; we support the preregistration badges and Registered Report format already provided by several journals [12]. Compliance can be monitored by journals in the review process. In the end, the most important stakeholder is the researcher, who can start to preregister right away. We aim to promote preregistration among researchers to increase awareness. To ease preregistration, we are focusing on minimising the administrative burden for researchers and offer online guidance and workshops.

## Abbreviations

Bf3R: German Centre for the Protection of Laboratory Animals
HARKing: hypothesising after results are known
UMCU: University Medical Center Utrecht
ZonMw: the Netherlands Organisation for Health Research and Development
